# Case report: Testicular manifestation of ANCA vasculitis

**DOI:** 10.1016/j.eucr.2025.102966

**Published:** 2025-01-30

**Authors:** Noah J. Sandel, Henry Wielzen

**Affiliations:** Department of Urology, Amsterdam UMC, University of Amsterdam, Amsterdam, the Netherlands

**Keywords:** ANCA vasculitis, Testicular malignancy, Testicular cancer, Vasculitis, Orchidectomy

## Abstract

ANCA-associated vasculitis is a rare autoimmune disorder affecting small to medium-sized vessels, often targeting the respiratory tract and kidneys. Testicular involvement is rare and can resemble malignancy, leading to unnecessary surgery. A 36-year-old male presented with painful fingers, oral and nasal ulcers, and knee arthritis. Elevated proteinase 3 (PR3) antibodies confirmed PR3 ANCA-associated vasculitis. During hospitalization, patient developed testicular pain, and an ultrasound raised suspicion of malignancy. An inguinal orchidectomy was performed, revealing inflammation consistent with vasculitis, but no malignancy. Testicular involvement in ANCA vasculitis can mimic cancer, and increased awareness may help prevent unnecessary surgical procedures.

## 1 Introduction

Antineutrophil cytoplasmic antibody (ANCA)-associated vasculitis is a systemic autoimmune disease characterized by inflammation and necrosis of small to medium-sized blood vessels, which can lead to multi-organ involvement (^1^ bbb,^2^). While it commonly affects the respiratory tract and kidneys, genital manifestations are rare and often underrecognized, especially when they mimic more common malignancies. Testicular involvement in ANCA-associated vasculitis is particularly uncommon, with clinical presentations that can be challenging to distinguish from testicular cancer, often leading to radical surgical interventions.^3^, ^4^, ^5^, ^6^ Here, we present a case of testicular manifestation of ANCA vasculitis that clinically resembled testicular cancer, resulting in an orchidectomy. This case report aims to raise awareness among medical specialists and addresses the importance of considering vasculitis in the differential diagnosis of testicular masses, potentially avoiding unnecessary surgical procedures in similar cases.

## 2 Case presentation

A 36-year old male patient with no significant prior medical history presented to the emergency department with painful, swollen and acrocyanotic fingers. Additionally, the patient exhibited oral and nasal ulcerative mucosal lesions, otitis media and arthritis of the knees. Internal medicine and rheumatology were consulted. Laboratory results revealed elevated proteinase 3 (PR3) antibodies (170 U/mL, normal range <3 U/mL), suggestive of PR3 ANCA-associated vasculitis, and the patient was admitted to the internal medicine department. There were no signs of respiratory or renal involvement.

**Fig. 1:**
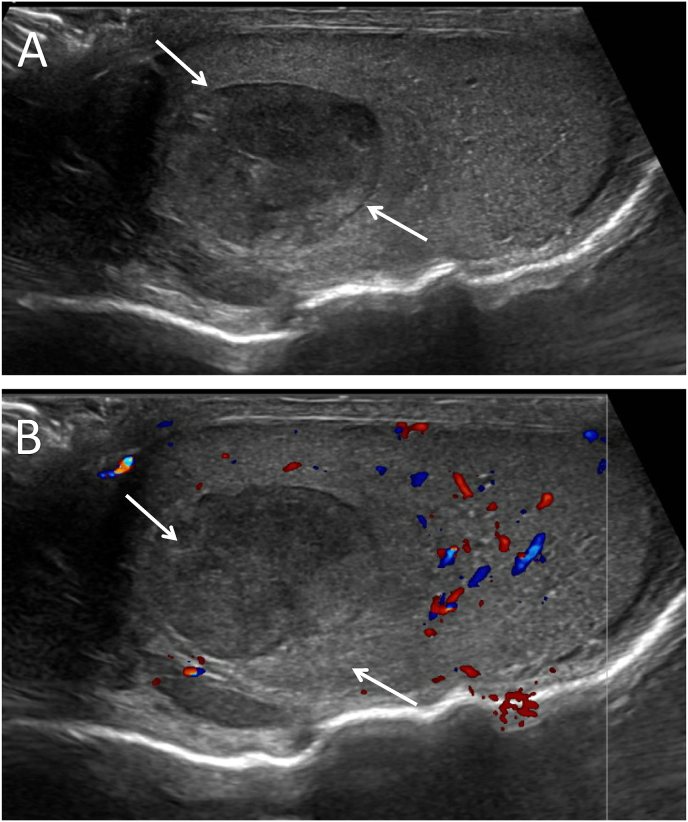
Ultrasound of right testicle showing a focal hypoechoic lesion in the right testicle (indicated by white arrows) (A), doppler ultrasound with no detectable blood flow in lesion (B).

With suspicion of PR3 ANCA-associated vasculitis, immunosuppressive treatment was initiated with methylprednisolone, rituximab and iloprost, alongside analgesics and nifedipine for vasodilation. During hospital admission, the patient reported mild pain in the right testicle and subsequently urology was consulted. At physical examination, a mass in the right testicle was palpated. Abdominal and pulmonal examination were without abnormalities. Serum markers, including alpha-fetoprotein, lactate dehydrogenase and human chorionic gonadotropin were within normal limits. Scrotal ultrasound revealed a focal hypoechoic lesion in the right testicle, measuring 1.9 × 1.4 cm, with no detectable blood flow (Fig. 1). As ultrasound could not rule out malignancy, an inguinal orchidectomy of the right testicle was performed on the following day. A subsequent CT scan showed no evidence of metastasis.

Pathology results showed active inflammation and necrosis consistent with ANCA vasculitis and revealed no signs of malignancy. The testicular mass contained hemorrhagic areas with fibrin deposition and granulocytic infiltration. Histological analysis of the small to medium-sized arteries revealed destruction of vessel walls with inflammation primarily involving neutrophils. The postoperative course was uneventful and patient was discharged home in consultation with internal medicine.

## 3 Discussion

Testicular manifestations of ANCA vasculitis are extremely rare, with clinical presentation which can be challenging to differentiate from testicular malignancies.

Anti-neutrophil cytoplasmic antibody (ANCA)-associated vasculitis is an auto-immune disease. ANCA are auto-antibodies that are important for neutrophils in fighting infections. ANCA normally binds to two proteins in the neutrophil, PR3 and myeloperoxidase (MPO). Patients with ANCA vasculitis have autoantibodies against PR3 or MPO resulting in a localized or systemic auto-immune reaction.^7^ Three subgroups of ANCA associated vasculitis are granulomatosis with polyangiitis (GPA), eosinophilic GPA (EGPA) and microscopic polyangiitis. Any tissue can be damaged by vasculitis, but the respiratory tract and kidneys are most commonly affected. In ANCA vasculitis neutrophils are activated which target healthy cells in vessel walls leading to tissue injury. Immunosuppressive medication is important for treatment. ANCA can be detected by laboratory tests on PR3 or MPO antibodies by immunofluorescence or ELISA or by a skin biopsy in case of cutaneous manifestations.

A case series described 16 patients with testicular manifestations of vasculitis.^5^ In 8 of these cases ultrasound images were available, that all revealed a circumscribed hypoechoic mass with a mean size of 2 cm, which is in accordance with our ultrasound results.

Another literature review included 72 patients with testicular vasculitis. Mean age at presentation was 42 years. Testicular pain was reported in 74 % of patients. Vasculitis involved the testicle in 80.3 % of cases, the epididymis in 44.6 %, and the spermatic cord in 30.6 %. Isolated testicular vasculitis was present in 51 % of patients, while 49 % had systemic vasculitis. Among the patients considered to have systemic vasculitis, final diagnoses were polyarteritis nodosa (PAN) in 62.9 % of patients, Granulomatosis with polyangiitis (GPA) in 17.1 % and Henoch-Schönlein purpura in 8.6 % of patients.^8^

Orchidectomy was more frequently performed in isolated testicular vasculitis than in systemic vasculitis (81.1 % vs. 42.9 %; p = 0.001).

Current imaging modalities are not able to distinguish isolated testicular manifestations of vasculitis from testicular malignancies. While orchidectomy is frequently performed in such cases, biopsy of the testicular mass can serve as a valuable diagnostic tool to confirm ANCA vasculitis, revealing characteristic features such as granulocytic infiltration and necrosis of small to medium-sized blood vessels, as demonstrated in our case. Histopathological evidence supporting ANCA vasculitis diagnosis can help avoid unnecessary surgery.

Alternatively, clinical observation may be considered for stable patients, with regular imaging and monitoring of laboratory markers such as PR3 antibodies to detect any progression. In case of progression, prompt surgical intervention can be pursued.

In all cases, a multidisciplinary approach involving rheumatology, urology and radiology is essential to ensure accurate diagnosis and appropriate management, ultimately reducing the risk of unnecessary orchidectomy and optimizing care for patients with testicular involvement in ANCA vasculitis.

## 4 Conclusion

Testicular localizations of vasculitis are extremely rare. In this case an inguinal orchidectomy was performed, as a testicular malignancy could not be ruled out. It is of importance of considering vasculitis in the differential diagnosis of testicular masses, potentially avoiding unnecessary surgical procedures.

## CRediT authorship contribution statement

**Noah J. Sandel:** Writing – original draft, Project administration, Methodology, Investigation, Data curation, Conceptualization. **Henry Wielzen:** Writing – review & editing, Resources, Conceptualization.

## Consent

Informed consent was obtained from the patient for publication of this case report in accordance with the journals patient consent policy.

## Ethical approval

The study conforms to recognized standards of World Medical Association Declaration of Helsinki.

## Data availability

Data used and/or analyzed from the current study are available from the corresponding authors per request.

## Funding statement

This research did not receive any specific grant from funding agencies in the public, commercial, or not-for-profit sectors.
